# Recent Advances and New Insights in Genome Analysis and Transcriptomic Approaches to Reveal Enzymes Associated with the Biosynthesis of Dendrobine-Type Sesquiterpenoid Alkaloids (DTSAs) from the Last Decade

**DOI:** 10.3390/molecules29163787

**Published:** 2024-08-10

**Authors:** Xu Qian, Surendra Sarsaiya, Yuanyuan Dong, Tuifan Yu, Jishuang Chen

**Affiliations:** 1 College of Biotechnology and Pharmaceutical Engineering, Nanjing Tech University, Nanjing 211800, China; 2 Bioresource Institute of Healthy Utilization, Zunyi Medical University, Zunyi 563000, China

**Keywords:** *Dendrobium* species, dendrobine-type sesquiterpenoid alkaloids, endophytes, biosynthetic pathway, intermediate compounds

## Abstract

*Dendrobium* species, which are perennial herbs widely distributed in tropical and subtropical regions, are notable for their therapeutic properties attributed to various bioactive compounds, including dendrobine-type sesquiterpenoid alkaloids (DTSAs). The objective of this review article is to provide a comprehensive overview of recent advances in the biosynthesis of DTSAs, including their extraction from *Dendrobium* species and endophytes, elucidation of associated genes through genomic and transcriptomic sequencing in both *Dendrobium* spp. and endophytes, exploration of the biosynthetic pathways of DTSAs, and drawing conclusions and outlining future perspectives in this field. Alkaloids, predominantly nitrogen-containing compounds found in medicinal orchids, include over 140 types discovered across more than 50 species. DTSAs, identified in 37 picrotoxane alkaloids, have a distinctive five-membered nitrogen heterocyclic ring. This review highlights endophytic fungi as alternative sources of DTSAs, emphasizing their potential in pharmaceutical applications when plant-derived compounds are scarce or complex. Genomic and transcriptomic sequencing of *Dendrobium* spp. and their endophytes has identified key genes involved in DTSAs biosynthesis, elucidating pathways such as the mevalonate (MVA) and 2-C-methyl-D-erythritol 4-phosphate (MEP) pathways. Genes encoding enzymes, such as acetyl-CoA C-acetyltransferase and diphosphomevalonate decarboxylase, are positively associated with dendrobine production. Despite significant advancements, the complexity of terpenoid biosynthesis in different subcellular compartments remains a challenge. Future research should focus on leveraging high-quality genomic data and omics technologies to further understand and manipulate the biosynthetic pathways of DTSAs and enhance their medicinal use.

## 1. Introduction

The orchid (*Dendrobium* sp.), a perennial herb, is widely distributed in tropical and subtropical regions. More than 28,000 species spanning 736 genera have been identified, as noted by Liu et al. [1]. According to “The Plants of the World”, over 80 *Dendrobium* species are found in China, predominantly south of the Qinling Mountains [2]. Numerous species from the *Dendrobium* genus, such as *Dendrobium nobile*, *D. huoshanense*, *D. chrysotoxum*, *D. fimbriatum*, and *D. officinale*, are integral to traditional Chinese medicine. Recent studies underline the varied therapeutic benefits of these species, including antioxidant, antitumor, anti-inflammatory, hypoglycemic, immune-boosting, and neuroprotective effects [3]. These medicinal properties are attributed to their myriad constituents, including polysaccharides, alkaloids, flavonoids, amino acids, bibenzyl, and several trace mineral elements. Notably, *D. nobile* is regarded as the fundamental species of *Shihu* in traditional medicine, with its annual production currently surpassing 4.5 million kg in China [1].

Alkaloids represent the most commonly occurring nitrogen-containing compounds found among medicinal orchids, serving as vital sources of bioactive compounds. Research has shown that over 50 orchid species produce more than 140 alkaloids. *Dendrobium* alkaloids, which possess complex chemical structures, represent the earliest identified category of compounds in *Dendrobium* [4]. These alkaloids encompass a diverse range of chemical constituents, including pyrrole, indolizidine, terpenoid alkaloids, organic amine alkaloids, indole, quinazoline, and others [5,6]. As with other genera of the Orchidaceae, this genus is notable for its indolizidine alkaloid and organic amine alkaloid content [7]. These chemicals are crucial for the pharmacological effects of the genus, positioning them as potential candidates for novel drug development. Of the alkaloids present in *D. nobile*, dendrobine was the first active compound identified in *D. nobile* and is considered the benchmark for both qualitative and quantitative assessments of the species, making up 92.6% of the alkaloids of *D. nobile* [8]. Since then, diverse sesquiterpenes and sesquiterpene alkaloids, including dendronobilin A [9], dendronobilin B [10], dendronobilin C [10], dendronobilin D [10], dendronobilin E [9], dendronobilin F [10], dendronobilin G [11], dendronobilin K [9], dendronobilin L [9], dendronobilin M [9], dendronobiloside A [11], dendronobiloside B [11], nobilomethylene [10], nobiline [12,13], (+)-(1R,5R,6S,8R,9R)-8,12-dihydroxy-copacamphan-3-en-2-one [14], (−)-(1S,2R,3S,4R,5S,6R,9S,12R)-3,11,13-trihydroxypicrotoxane-2(15)-lactone [15], and (+)-(1R,2S,3R,4S,5R,6S,9R)-2,11,12-trihydroxypicrotoxane-3(15)-lactone [14] derived from picrotoxane and dendrobine lactone have been isolated from *D. nobile*.

Extraction from *Dendrobium* plants is a prevalent method for obtaining natural DTSAs. Although previous studies have enhanced extraction efficiency, large-scale DTSAs production faces significant challenges [5]. The primary hurdle is the scarcity of plant materials, caused by the slow growth of these plants and the low DTSA content within them. Additionally, the extraction process is complex due to the similar structures of co-existing alkaloids, complicating their separation. Furthermore, excessive harvesting of plants depletes soil nutrients and harms the environment. Consequently, the slow-growing nature of *Dendrobium* plants has shifted focus towards in vitro reproduction methods.

Several groups have reviewed and summarized the process of *Dendrobium* propagation, including protocols for breeding techniques based on tissue culture and bioreactor technology, mass propagation, biotechnology, molecular aspects, breeding of orchid protocorm-like bodies, and biotechnological applications in the orchid industry [16,17,18]. Furthermore, chemical synthesis has been implemented as a supplementary method, alongside plant extraction and tissue culture strategies. Nevertheless, this approach is not without its limitations. These include the potential for complex multi-step reactions, low yields, and the possibility of environmental pollution. Several reviews have summarized research on the chemosynthesis methods of picrotoxane sesquiterpenoids and alkaloids [3,19,20]. 

**Figure 1:**
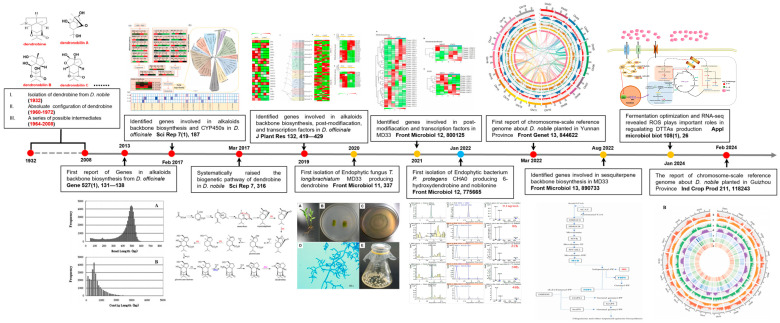
Brief timeline of some key milestones during the research history of DTSAs. Notable studies are included and key references for each study are provided. The red, yellow, and blue cycles represent studies on *Dendrobium* species [21,22,23,24,25,26], *T. longibrachiatum* MD33 [27,28,29,30], and *P. protegens* CHA0 [2], respectively.

**Figure 2:**
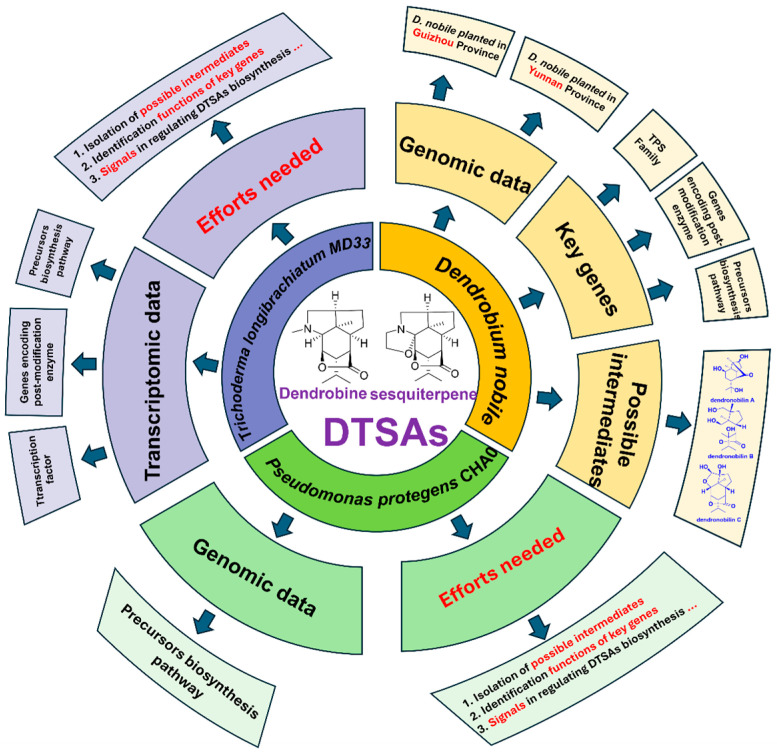
An overview of available data for complete biosynthesis of DTSAs.

Consequently, bioproduction presents a viable alternative to chemical synthesis, spurred by the exploration of plant endophytes which has captivated scientific interest in deciphering the biosynthetic pathways of DTSAs (Figure 1). While the enzymes implicated in DTSAs biosynthesis are not yet completely deciphered, extensive research continues. Additionally, the heterologous expression of genes involved in DTSAs synthesis poses significant challenges. Hence, an improved grasp of the structures and catalytic mechanisms of the key enzymes is crucial. This review aims to outline the synthesis of DTSAs, highlighting the isolated compounds from both *Dendrobium* species and endophytes, as well as the genomic and transcriptomic approaches used to identify putative biosynthetic genes and regulators. Moreover, it describes the DTSAs biosynthesis pathway in *Dendrobium* species and endophytes, aiming to shed light on the entire biosynthetic process of DTSAs (Figure 2).

## 2. The Resources for Obtaining Dendrobine-Type Sesquiterpenoid Alkaloids (DTSAs)

### 2.1. Dendrobine-Type Sesquiterpenoid Alkaloids (DTSAs) Extracted from Dendrobium Species 

**Figure 3:**
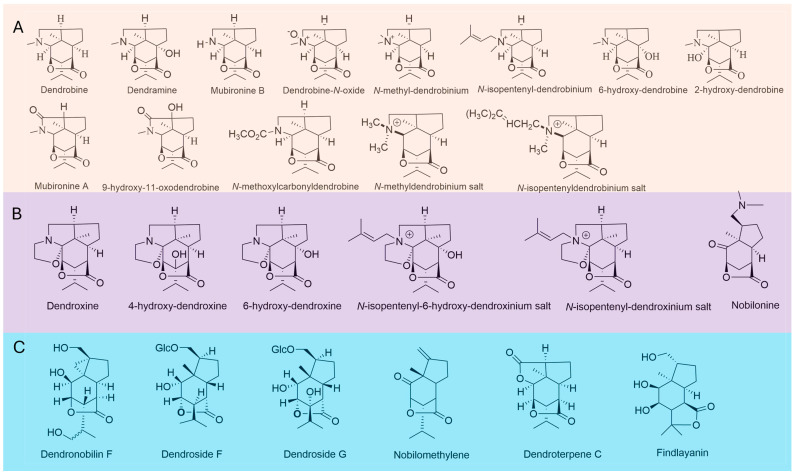
Chemical structures of alkaloids identified in *Dendrobium* species. (**A**) Dendrobine-type alkaloids and *N*-dendrobinium salts. (**B**) Dendroxine-type alkaloids. (**C**) Dendrobine-type sesquiterpenes.

Currently, 37 picrotoxane alkaloids have been isolated, identified, and classified into distinct categories, including dendrobine, nobilonine, dendrine, dendroxine, and seco-dendrobine (Figure 3) [8]. Characteristically, most of these alkaloids incorporate a nitrogen atom at the C-11 position within the picrotoxane sesquiterpene framework, and notably, none feature an epoxide ring [3,5]. The principal structural feature of dendrobine alkaloids is the presence of a five-membered nitrogen heterocyclic ring situated between C-2 and C-9. Shi et al. systematically reviewed the structures, chemical synthesis methods, and biosynthesis pathways of these alkaloids [3]. Dendrobine, the first picrotoxane sesquiterpene alkaloid to be isolated from *D. nobile* in 1932, stands out as a notable example among picrotoxane alkaloids [4]. Subsequent to 1960, several Japanese research teams succeeded in elucidating the absolute structure of dendrobine through chemical degradation, feasible spectroscopic analyses, CD spectroscopy, and applying the octant rule [31,32,33,34]. Since then, several dendrobine-derived compounds have been identified using various tools. For example, dendramine, a 6-hydroxylated analog of dendrobine, was initially isolated from *D. nobile*. The complete structure was determined by a combination of degradation reactions and spectroscopic analysis [35,36,37]. The structure of mubironine A was deduced by 2D NMR spectroscopy as 11-oxodendrobine [38]. Prior to the isolation of mubironine A, the isolation of 9-hydroxy-11-oxodendrobine was performed. The structure of this compound was confirmed by the analysis of X-ray single crystal diffraction data, which indicated the existence of an additional hydroxyl group at C-9 compared to that of mubironine A [39]. Mubironine B was initially isolated and characterized as an N-demethyl dendrobine [38]. More information on spectral characteristics of picrotoxane alkaloids can be found in Wang’s review article [40].

Recently, the application of UPLC-QTOF-MS has significantly enhanced the resolution, sensitivity, and throughput of metabolite analysis. Moreover, Mass spectrometry imaging (MSI), particularly matrix-assisted laser desorption/ionization mass spectrometry imaging (MALDI-MSI), has proved effective for visualizing metabolites in plant tissues [41,42,43]. Using both Ultra-Performance Liquid Chromatography/Quadrupole Time-of-Flight Mass Spectrometry (UPLC-QTOF-MS) and Matrix-Assisted Laser Desorption/Ionization Time-of-Flight Mass Spectrometry Imaging (MALDI-TOF-MSI), researchers have been able to map and visualize the spatial distributions of alkaloids and sesquiterpenoids [1]. Subsequent analyses revealed that these alkaloids are predominantly distributed in the parenchyma or vascular bundles, with dendrobine being particularly concentrated in the epidermis, prominent in the vascular bundle, and scarce in the parenchyma, indicating varied sites of dendrobine biosynthesis within different organs. 

### 2.2. Dendrobine-Type Sesquiterpenoid Alkaloids (DTSAs) Extracted from Endophytes

Endophytic fungi produce an extensive range of biologically active substances. They are able, in some cases, to synthesize compounds that are similar in their pharmacological activity to the substances identified in plants [44]. Particularly valuable when the desired bioactive secondary metabolites are not commercially accessible, originate from slow-growing, rare, or endangered plants, or are challenging to synthesize due to their substantial molecular weight or complex structure, endophytic fungi are a feasible solution. The secondary metabolites produced by these fungi have garnered significant interest, especially from researchers focusing on medicinal plants, mangroves, and marine microorganisms.

Following the discovery of paclitaxel-producing endophytic fungi, researchers have been increasingly motivated to identify additional endophytes capable of synthesizing unique compounds that help safeguard plant species from extinction. To date, there have been few reports on the isolation of DTSA-producing endophytes. The strain *Trichoderma longibrachiatum* MD33 with the ability to produce dendrobine [27] was isolated from the stem of *D. nobile*, and its structure was detected by ^1^H and ^13^C NMR spectroscopy [45]. *Pseudomonas protegens* CHA0, an endophytic bacterium, was also isolated from the *Dendrobium* stem, with the ability to produce two dendrobine-type sesquiterpenoid alkaloids [2]. The two alkaloids showed antimicrobial activity, and their structures were determined by UPLC-QTOF-MS.

The composition of endophytic fungi and bacteria in medicinal plants is influenced by several factors, including habitat, age gradient, and cultivation methods. These elements are particularly critical in determining the endophytic fungal composition of *Dendrobium* species. Research by Xu and colleagues has demonstrated that variations in the endophytic fungal community significantly impact dendrobine biosynthesis in *D. nobile* across four different habitats. Specially, differences in altitude influenced the relative abundances of endophytes, with a notable correlation between the altitude changes and the relative abundance of *Toxicoladosporium* in Guizhou Province, which in turn significantly correlated with variations in dendrobine content. Research, including that by He et al., has extensively analyzed endophytic fungal communities in *D. nobile* of varying ages and their correlation with dendrobine content [46]. The study found that the abundance of endophytes like *Gibberella*, *Basidiomycota*, *Cyphellophora*, and *Glomerella*, had either positive or negative correlations with dendrobine content. Additionally, a significant change in fungal community composition in *D. nobile* stems was evident across an age gradient. For instance, one-year-old plants were predominantly inhabited by *Olipidium*, *Hannaella*, and *Plectospherella*, whereas two-year-old plants were primarily associated with *Strelitziana* and *Trichomerium*. In contrast, three-year-old plants showed a greater diversity of endophytic fungi, including the genus *Rhizopus*. This research suggested a potential mechanism by which endophytic fungi may influence the transcriptome of the stem and influence dendrobine synthesis in *D. nobile*, possibly via altering plant hormone signaling and alkaloid biosynthesis pathways [47]. The impact of diverse cultivation modes (living tree epiphytic, cliff epiphytic, and pot) and plant organs (leaf, stem, and root) on microbial communities was comprehensively evaluated by Wu’s group using high-throughput sequencing techniques. Their investigation revealed significant variations in microbial communities across plants and substrates, contingent upon the specific cultivation mode [48]. 

Endophytic bacteria, unlike their fungal counterparts, have received relatively less attention. In a study conducted by Zhao’s group using 16S rRNA high-throughput sequencing, various organs (roots, stems, and leaves) of *D. nobile* were analyzed. The study identified 24 bacterial phyla and 448 genera of endophytes, with *Proteobacteria*, *Actinobacteria*, and *Acidobacteria* emerging as predominant phyla. The roots exhibited higher relative abundance and diversity of endophytes compared to the stems and leaves. Most endophytes appear to play roles in nutrient metabolism, and certain bacterial taxa seem to facilitate the recruitment of other genera within the same phylum [49]. Additionally, the endophytic bacterial community in the stems of *D. huoshanense*, *D. moniliforme*, and *D. officinale* are influenced by their origins and cultivars [50]. These findings highlight the complex role of endophytes in regulating the synthesis and accumulation of dendrobine in *D. nobile*, as revealed through high-throughput sequencing.

### 2.3. Various Strategies to Increase DTSAs Production in Endophytes

Although different endophytes producing DTSAs have been reported, the content within endophytes is still low and insufficient to meet the requirements of industrial production. In order to increase DTSAs production, some methods have been reported in recent years by controlling the composition of fermentation medium, the supply of precursors, and abiotic elicitors. Qian et al. used response surface methodology to change the amount of glucose, beef extract, and CoCl_2_, and finally increased the yield of DTSAs from *T. longibrachiatum* MD33 [28]. The transcriptomic analysis of strains treated with CoCl_2_ revealed that reactive oxygen species (ROS) have a signal transcription function regulating DTSAs biosynthesis. The production of dendrobine increased by 44.6% when 20 μmol/L methyl jasmonate was added in the fermentation medium [29]. In another study, promising results were observed when *D. nobile* seedlings were co-cultured with *T. longibrachiatum* MD33, with dendrobine production increasing by 9.7 times [45], suggesting that dendrobine is produced by endophytic fungi in conjunction with a native host. 

## 3. Genomics and Transcriptomics Used to Elucidate Associated Genes 

### 3.1. Genes Identified in Dendrobium spp. through Genomic and Transcriptomic Sequencing 

In recent decades, the advent of DNA sequencing and the concomitant development of molecular identification technologies have paved the way for the pervasive application of various omics technologies, including genomics, transcriptomics, proteomics, and metabolomics. These tools have been employed extensively in elucidating the physiological and molecular mechanisms that drive the biosynthesis of bioactive compounds. Genomic data provide essential and in-depth understanding of the genetic background of medicinal plants, effectively linking genetic bases to the synthesis of active compounds. To date, the genomic data of *D. nobile* planted in Yunnan and Guizhou Province have been reported (Table 1), with genome size around 1.19 Gb and heterozygosity of 1.35% and 2.03%, respectively [21,22]. A comparison of the two is presented in Table 2. Both versions were assembled at the chromosome level using third-generation and Hi-C sequencing platforms. The number of protein-coding genes ranged from 27,765 to 30,828. Variations in genomic data between the two versions may arise from differences in assembly strategies and genome annotation approaches. Analysis of whole-genome duplication (WGD) revealed that *D. nobile* from Yunnan Province underwent two WGD events, whereas *D. nobile* from Guizhou Province did not experience a unique WGD event. Instead, it shares an ancient WGD event with *D. huoshanense* and *D. chrysotoxum*.

A primary aspect of research within medicinal plant genomics pertains to the annotation and detection of candidate genes crucial for the biosynthesis of active compounds. Analysis of the two versions of the *D. nobile* genome revealed significant expansion of certain genes encoding enzymes critical for the production of these compounds. Specially, using the genomic data version from Yunnan province, researchers have comprehensively identified 306 genes associated with the biosynthesis pathways of polysaccharides, alkaloids, and dendrobine [22]. For alkaloids biosynthesis, 20 genes were identified as potentially involved. Aside from the *DHS*, *DXS*, and *HMGR* gene families, most genes were single-copy genes. As dendrobine is the dominant alkaloid in *D. nobile*, genes involved in backbone formation and modification were systematically analyzed. In detail, 51 TPS genes were identified and classified into TPS-a subfamily, TPS-b subfamily, TPS-c subfamily, TPS-e/f subfamily, and TPS-g subfamily. Notably, the TPS-a subfamily, which uniquely encodes sesqui-TPSs, comprised 21 genes in *D. nobile*, a number that is greater than that observed in other *Dendrobium* species. Given that the TPS-a subfamily exclusively encodes sesqui-TPSs [51], the presence of a larger number of TPS-a genes in *D. nobile* may be a significant contributor for the higher production of dendrobine. There were 228 CYP450 genes, mainly distributed in the CYP85 clans. The genomic data of *D. nobile* from Guizhou Province was reported by Zhao’s lab, with a higher degree of completeness, including a scaffold N50 of 61.81 Mb and a contig N50 of 10.01 Mb. The key genes in picrotoxane-type skeleton biosynthesis, the TPS gene family, and the CYP450 gene family were dissected [21]. During the biosynthesis of the picrotoxane-type skeleton, researchers identified 22 key enzyme-encoding genes, including acetyl-CoA C-acetyltransferase (AACT), diphosphomevalonate decarboxylase (MVD), and farnesyl pyrophosphate synthase (FPPS). Analysis of gene expression across different tissues, developmental stages from one-year seedlings, and seedlings either infected or uninfected with its mycorrhizal fungus MF23 (*Mycena* sp.) demonstrated that the sesquiterpenoid backbone of picrotoxane-type sesquiterpenoid alkaloids (PSAs) predominantly relies on the MEP pathway. 

Following the application of protein domain and phylogenetic analyses, 64 members belonging to the TPS gene family were identified. The TPS-a subfamily represents the primary enzymatic group involved in dendrobine backbone biosynthesis [22,52], with 33 TPS genes ascribed to this subfamily. Comparative transcriptome analysis indicated that eight genes (*TPS2*, *TPS24*, *TPS35*, *TPS32*, *TPS38*, *TPS42*, and *TPS43*) likely play roles in the biosynthesis of the sesquiterpene skeleton of dendrobine. Further research also uncovered that various members from 71 clans of the CYP450 gene family can catalyze reactions using the sesquiterpene nucleus as a substrate [53,54,55]. Following a comprehensive analysis of the data, 148 constituents belong to 71 clans of the CYP450 gene superfamily were identified. A total of 148 *CYP450* genes were grouped into 15 distinct subfamilies, and 22 of these genes, namely *CYP71K6*, *CYP71K10*, *CYP71P1*, *CYP75B2*, *CYP78A5*, *CYP76AD2*, *CYP76AD6*, *CYP706A1*, *CYP81DL1*, *CYP89B1*, *CYP71W2*, *CYP71K14*, *CYP71K17*, *CYP71K35*, *CYP701A1*, *CYP707A2*, *CYP76AD10*, *CYP74A2*, *CYP87A1*, and *CYP87A3* exhibited a correlation with dendrobine content under various treatment conditions. Xu’s lab also systematically identified CYP gene family members in the genomes of four medicinal *Dendrobium* species and analyzed their motif composition. In *D. nobile* genome data (GenBank accession number PRJNA725550), 226 P450 genes belonging to eight clans (clans 51, 71, 72, 85, 74, 86, 97, and 711) were obtained. Two genes, *DnoNEW43* and *DnoNEW50*, have been proposed to play a role in the synthesis of alkaloids in *D. nobile* [56].

Prior to the release of genomic data, various transcriptomic studies had been conducted on the alkaloid biosynthesis pathway in *Dendrobium* species (Figure 2). For instance, Guo et al. were pioneers in utilizing the Roche 454 GS FLX Titanium platform to identify and document 25 genes linked to the construction of alkaloid backbones [23]. In the case of *D. nobile* infected by mycorrhizal fungus MF23, comprehensive transcriptome analysis led to the identification of 16 genes associated with dendrobine backbone biosynthesis [24]. The increase of dendrobine content might be associated with the formation of pelotons, which is a sign of the establishment of typical orchid mycorrhizae and considered as a nutritional pool or transformation center to supply nutrition [24,57,58]. These findings highlighted the significant role of the mevalonate (MVA) pathway in this process. The preliminary biosynthetic steps for dendrobine involve both the MVA and the methylerythritol phosphate (MEP) pathways. These pathways are crucial as they both contribute to forming isopentenyl diphosphate (IPP), a precursor for terpenoid alkaloids. Notable enzymes active in the MEP pathway, like 1-deoxy-D-xylulose-5-phosphate (DXS) and 1-deoxy-D-xylulose-5-phosphate reductoisomerase (DXR), and those in the MVA pathway, including 3-hydroxy-3-methylglutaryl-CoA synthase (HMGS) and 3-hydroxy-3-methylglutaryl-coenzyme A reductase (HMGR), have been annotated in *D. officinale* [25]. Integrating transcriptome and metabolome analysis offers a more comprehensive exploration of the mechanism. To investigate the variations and genetic mechanism across different epiphytic patterns, Xu’s group constructed a full-length transcriptome database for the whole organs and tissues of *D. nobile* throughout its growth cycle [59]. This led to the identification of 387 distinct genes, which correspond to 66 different metabolites involved in processes such as flavonoid metabolism, purine metabolism, and terpenoid backbone biosynthesis. For cultivation mode, the epiphytic patterns of Danxia stone are the most suitable based on the accumulation of metabolites.

Post-modification enzymes such as cytochrome P450s (CYP450s), which mediate oxidation and hydroxylation reactions, significantly enhance the diversity of DTSAs. Recognized as a diverse superfamily of monooxygenases, CYP450s are crucial for the biosynthesis of specialized metabolites, and a number have been isolated and characterized [60]. For instance, analysis of 454 EST sequences against the SwissProt database revealed 93 CYP450s transcripts in *D. officinale*, spanning 17 families [23]. Notably, transcripts from the CYP71 family appear particularly involved in the hydroxylation steps of alkaloid biosynthesis. Further, various post-modification enzymes integral to dendrobine’s biosynthetic pathway, including CYP450s, aminotransferase, and methyltransferase, have been identified in both *D. nobile* [24] and *D. officinale* [25]. Specific enzymes such as CYP1D10, METTL23, ATX4, and BCAT2 were markedly upregulated following MF23 infection [24]. Recently, Zhao et al. demonstrated that transient expression of genes including *CMK*, *DXR*, *MCT*, *STR1*, *CYP*94*C*1, *BCAT*2, and *METTL*23 in *D. catenatum* leaves resulted in a two-fold higher increase in dendrobine yield than that of the empty vector control [61]. Therefore, these genes are likely to play substantial roles in enhancing dendrobine biosynthesis. During the biosynthesis of DTSAs, sesquiterpene glycosylation represents a crucial process, sharing pathways upstream with alkaloids synthesis. Systematic studies were conducted by He’s group on this modification process. Initially, they established a quantitative analysis method for sesquiterpene glycosides (SGs) including dendronobiloside E, dendromoniliside D, and others using high-performance liquid chromatography coupled with triple quadrupole tandem mass spectrometry (HPLC-QqQ-MS/MS) in multiple reaction monitoring (MRM) mode. Their findings revealed that the primary factors affecting SGs contents were age, geographical origin, altitude, and epiphytic pattern [62]. Interestingly, while the content of alkaloids decreased over time, that of glycosides increased [63]. Following this, a comparative transcriptomic analysis was performed on 1- and 3-year-old *D. nobile*, identifying approximately 184 UDP-glycosyltransferase genes. Two of these genes were highlighted as potentially playing a key role in sesquiterpenes glycosylation [64]. The involvement of UDP-glycosyltransferases in sesquiterpene glycosylation was further confirmed by a combination of metabolomic and transcriptomic methods, alongside RT-PCR and molecular docking techniques.

Transcription factors (TFs) are crucial in regulating the expression of genes involved in alkaloid biosynthesis, thereby affecting the composition of active compounds in higher plants. Li et al. utilized WGCNA and co-expression correlation analysis to investigate the distribution of TFs in *D. nobile*. Key TFs, including *ATHB-13*, *ATHB-13–1*, *MADS16*, *MADS16–1*, *GT-1*, *IPN2*, *MYB30*, *MYB101*, and *ERF109*, were identified as central to promoting dendrobine accumulation by regulating structural genes (*AACT*, *CMK*, *MCS*, *HDR*, *MVD*, *IDI2*, *DXS1*, and *FDPS)* in the terpenoid skeleton biosynthesis pathway. In a separate study, Yuan et al. identified 570 putative TF genes across 18 major TF families in *D. officinale* [25]. The majority of TFs from the NAC, WRKY, bHLH, MYB, and MYB-like families showed upregulation under MeJA treatment. Notably, the overexpression of *MYB61* contributed to a more than two-fold increase in dendrobine production, underscoring the significant role of the MYB family in boosting dendrobine synthesis.

### 3.2. Genes Identified in Endophytes through Genomic and Transcriptomic Sequencing

Although fungal and bacterial endophytes can produce DTSAs, their biosynthetic genes and enzymes remain largely unexplored. Research involving genome and transcriptome analyses of *Dendrobium* plants provides a foundational framework for understanding the biosynthetic steps of DTSAs in endophytes. This process encompasses three primary stages: IPP formation, sesquiterpene skeleton construction, and post-modification [1,24,65]. Following the addition of methyl jasmonate, transcriptomic analyses identified differential expression in several MVA pathway genes, including isopentenyl-diphosphate delta-isomerase, iphosphomevalonate decarboxylase, and farnesyl diphosphate synthase, indicating that MD33 may synthesize dendrobine via the MVA pathway (Table 1) [29]. Additionally, comparative transcriptomic analysis has identified several post-modification enzymes [28,30]. Detailed analysis identified four putative genes (*Cluster-1146.0*, *Cluster-4183.0*, *Cluster-5186.0*, and *Cluster-5325.0*) from four clans (CYP531, CYP62, CYP53, and CYP507 clan) of the Cytochrome P450 family. Clustering analysis revealed that the expression of a gene encoding branched amino acid aminotransferase increased by approximately 1.98-fold, a finding also supported by the transcriptomic analysis following cobalt chloride induction [28]. Furthermore, 16 putative methyltransferases were identified, with half displaying heightened expression. These findings suggested that the DTSA biosynthetic pathway may be conserved between *Dendrobium* plants and endophytic fungi. Recent investigations into endophytic bacteria, spurred by their genomic isolation, are examining their synthetic pathways. After completing whole-genome sequencing, the involvement of genes in the MEP pathway within *P. protegens* CHA0 was established, but genes associated with the downstream biosynthetic pathways of DTSAs are yet to be explored.

## 4. The Biosynthetic Pathways of DTSAs

**Figure 4:**
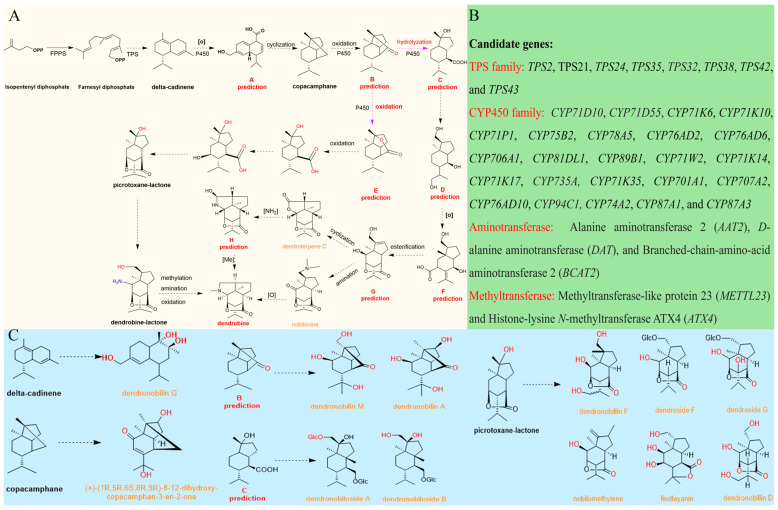
Outline of the DTSAs biosynthetic pathway in *Dendrobium* species. (**A**) The proposed biosynthetic process of DTSAs. (**B**) Candidate genes involved in the backbone formation and post-modification were obtained from genomic and transcriptomic data of *D. nobile*. (**C**) The potential backbone structures that form intermediates. The orange labels indicate possible intermediates that have been isolated and identified, which may participate in this biosynthetic process. The dashed lines represent the hypothetical processes. The mauve dashed lines mean putative ways of forming intermediates through either oxidation or hydrolysis. The red letters of the functional groups are transitory putative modifications, representing the difference between intermediates with their potential backbone structures.

In light of the recent publication of genomic data for *D. nobile* and previous hypotheses regarding the biosynthesis of DTSAs, as well as the identification of possible intermediates extracted from *D. nobile*, the biosynthetic process of DTSAs has been further elucidated through a series of speculations. The pathways supplying precursors, especially mevalonate (MVA) and 2-C-methyl-D-erythritol 4 phosphate (MEP) pathways, are well documented in the context of DTSAs biosynthesis. These pathways initially produce isopentenyl diphosphate (IPP), which is then converted into farnesyl diphosphate (FPP) by farnesyl phosphate synthase (FPPS), serving as a critical upstream process. In the MVA pathway, key enzyme-coding genes such as acetyl-CoA C-acetyltransferase (*AACT*), phosphomevalonate kinase (*PMK*) kinase, and diphosphomevalonate decarboxylase (*MVD*), along with 1-deoxy-D-xylulose-5-phosphate synthase (*DXS*) and 1-deoxy-D-xylulose-5-phosphate reductoisomerase (*DXR*) from the MEP pathway, have been identified as positively influencing dendrobine production [3,8]. In addition, recent studies have also highlighted the role of transcription factors, including *ATHB-13*, *ATHB-13–1*, *MADS16*, *MADS16–1*, *GT-1*, *IPN2*, *MYB30*, *MYB101*, and *ERF109*, in regulating precursor synthesis pathway [21]. The most recent advancements of backbone formation and modification were depicted in Figure 4A. More information on candidate genes and intermediates is illustrated in Figure 4B,C. In detail, the products from the MVA and MEP pathways, IPP and its isomeric DMAPP, are connected together by farnesyl phosphate synthase to generate farnesyl pyrophosphate (FPP). Subsequently, this compound undergoes cyclization, catalyzed by terpene synthase (TPS), to form delta-cadinene through intramolecular reaction. The intermediate dendronobilin G may be derived from this structure (Figure 4C). In total, 21 genes from the TPS-a family were identified and presumed to participate in this process based on the genomic and transcriptomic data. After that, the intermediate Dendronobilin G is predicted to form intermediate A as a consequence of the action of P450 oxidoreductase. The rearrangement (C2 and C7) cyclization of the A skeleton molecule led to the formation of copacamphane, with the compound (+)-(1R,5R,6S,8R,9R)-8,12-dihydroxy-copacamphan-3-en-2-one being deemed an appropriate modification based on this structure configuration (Figure 4C). The compound copacamphane was further oxidized under the function of CYP450 to form B skeleton. Recently, it was pointed out that B skeleton can transform into either compound C or E through the catalysis of CYP450 enzymes. Compound C is formed through ring opening and hydrolysis, which establishes the structural basis for dendronobiloside A and dendronobiloside B. In the process of obtaining a skeletal structure resembling picrotoxane-lactone, compound D undergoes additional redox reactions to give rise to compound F, which later undergoes esterification to yield compound G. Compound E requires catalysis by CYP450 enzymes through several steps. During this process, there are about eight clans of CYP450s which have been identified, including clan51, clan74, clan 711, clan 97, clan 71, clan 72, clan 85, and clan 86 [23]. To obtain the final structure of dendrobine, picrotoxane-type sesquiterpenes undergo several pathways, one of which includes the formation of dendroterpene C, which is then aminated to form compound H, and subsequently methylated to form dendrobine. Alternatively, these sesquiterpenes might directly undergo amination to produce nobiline, followed by cyclization and decarboxylation to form dendrobine. Another route involves compound E initially forming dendrobine-lactone, which then undergoes oxidation, amination and methylation to ultimately produce dendrobine.

**Figure 5:**
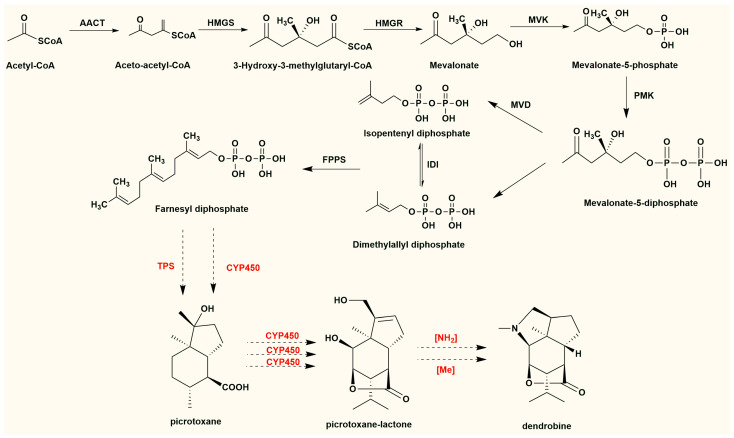
The putative biosynthetic process of DTSAs in endophytic fungus. The enzymes in red are involved in backbone formation and post-modification. The dashed lines represent hypothetical processes requiring multiple steps.

Based on the transcriptomic data published on dendrobine biosynthesized in *Dendrobium* species and endophytic fungus, the biosynthesis process of endophytic fungi was also proposed to occur in three steps (Figure 5): biosynthesis of the sesquiterpene skeleton, formation of the picrotoxane skeleton, and post-modification of the skeleton. In general, mevalonate (MVA) is considered the main upstream contributor to the DTSAs biosynthetic pathway for the synthesis of IPP [29]. After generating farnesyl pyrophosphate (FPP), it is cyclized via intramolecular cyclization under the action of terpene synthase (TPS). To obtain a picrotoxane skeleton, a series of cytochrome oxidases (CYP450s) may participate in skeleton modification. Aminotransferases and methyltransferases may be key enzymes that contribute to the final formation of dendrobine. The genes encoding the enzymes involved in post-modification need to be confirmed in the future.

## 5. Conclusions and Outlook

Over the past few years, advances in sequencing technology and various “omics” technologies have greatly facilitated the functional identification of previously unknown genes associated with specific plant metabolites. The achievement of high-quality genome sequencing and re-sequencing offers substantial opportunities for exploring metabolic pathways in crops and valuable traditional Chinese medicinal plants. Despite these advances, the complex diversity of metabolic and regulatory networks poses significant challenges to terpenoid engineering in plants. Notably, the synthesis of plant secondary metabolites significantly varies across different tissues, posing a substantial challenge for gene identification. For instance, taxol synthesis predominantly occurs in the bark of trees, where the enzymes crucial for its biosynthesis are highly expressed [66]. Similarly, flavonoids like quercetin, known for their antioxidant properties, are mainly produced in the skin of apples. The enzymes catalyzing the flavonoid biosynthetic pathway primarily reside in the fruit’s epidermal cells, contributing to peel color and UV protection [67]. Analysis of the spatial distribution of alkaloids within *D. nobile* highlighted that dendrobine was especially abundant in the epidermis and the vascular bundle, but less abundant in the parenchyma. Consequently, it is necessary to confirm whether genes related to dendrobine biosynthesis are also distributed in different tissues.

Identifying and screening enzymes critical for synthesizing active compounds in plants are essential for deciphering and potentially modifying plant metabolic pathways. In order to elucidate the biosynthesis pathway of DTSAs, it is necessary to follow the outlined steps. First, large datasets from RNA-seq experiments should be combined to build a BLAST library. Bioinformatic analyses, including differential expression and co-expression network analyses, such as WGCNA, are crucial for identifying potential enzyme-coding genes. Second, once candidate genes are highlighted, predictive enzymology can be used to speculate on possible biochemical functions. This can be achieved using databases and software to anticipate enzyme activity based on known functions of homologous sequences. Tools, such as Blast2GO and KEGG pathway mapping, play key roles in these predictions. Third, confirming enzyme expression within specific tissues involves techniques such as Western blotting or immunolocalization, which directly detects the presence and distribution of enzyme proteins. This confirms the tissue-specific expression observed at the transcriptomic level. For additional validation, the enzymatic activity of these proteins must be ascertained. This is achieved by expressing candidate enzymes in vitro (using systems such as bacteria or yeast) and evaluating their activity. By analyzing the reactions they catalyze, the roles of these enzymes in specific biosynthetic pathways can be verified. Deepening functional characterization involves genetically modifying the plant to either knock out or overexpress candidate genes using tools such as RNAi or CRISPR/Cas9. Finally, connecting enzymatic activity to tangible changes in metabolite profiles requires metabolomic studies. Techniques like GC-MS or LC-MS are utilized to examine the impact of altered enzyme expression on the plant’s metabolome, specifically focusing on the concentrations of targeted active compounds.

Despite efforts to isolate endophytes, co-cultivate endophytic fungi with host plants, optimize fermentation conditions, and utilize irradiation-assisted production enhancement, there have been limited breakthroughs in understanding the interaction between plants and their endophytic fungi [28,30,68]. Research on the biosynthesis of DTSAs in endophytes faces significant challenges. Isolated endophytic fungi often exhibit low viability and effective preservation techniques remain undeveloped. Frequent cultivation passaging contributes to suboptimal DTSA production levels. Currently, the cultivation of endophytic fungi is limited to laboratory settings, which restricts the large-scale production of DTSAs. Additionally, most enzymes hypothesized to be involved in the post-modification stages of DTSA biosynthesis have been identified through transcriptomic data, yet verification experiments both in vitro and in vivo are lacking. For instance, although genes such as *DnFPPS*, *HMGS*, *HMGR*, and *MVD* have been cloned, characterized, and expressed in *Dendrobium* species [69], the roles of other predicted enzyme genes remain undefined in both plants and fungi. Conducting in vivo experiments necessitates the development of genetic transformation systems for endophytic fungi, which would allow for validation of the proposed biosynthesis pathways and potentially enhance DTSA production. Furthermore, isolation and analysis of potential intermediates in endophytes are crucial for confirming the functions and processes of post-modification enzymes.

The identification of the genes responsible for these biochemical pathways is a fundamental step, but equally crucial is the enhancement of their production to meet the demands of industrial-scale manufacturing. In this context, both abiotic and biotic elicitors have been demonstrated to be effective in increasing the yield of active substances. Nevertheless, the precise nature of the signal transduction pathways that mediate these enhancements remains unclear, necessitating further investigation. Recent studies have shed light on the role of reactive oxygen species (ROS), calcium ions (Ca^2+^), and nitric oxide (NO) as key signaling molecules in the regulation of secondary metabolism [70,71,72,73,74]. To facilitate large-scale production of these compounds, several initiatives are essential. Firstly, the application of advanced omics technologies and bioinformatics is critical for delineating these signaling pathways. Secondly, employing synthetic biology to modify endophytic fungi could lead to strains with improved production capabilities under enhanced signaling pathways or novel pathways being introduced. Thirdly, the integration of bioreactor technology and metabolic engineering could provide the requisite controlled environments to achieve this scale-up. In summary, the sophisticated regulation by signaling molecules over the biosynthesis of DTSAs in endophytes is a promising area of research that merges basic science with industrial potential. As our comprehension of the biosynthetic pathways and signal transduction networks is deepened by various endeavors, innovative techniques to promote the sustainable biosynthesis of DTSAs will be pushed forward.

## Figures and Tables

**Table 1:** Genomic and transcriptomic sequencing of *Dendrobium* spp. and endophytes revealing genes related to DTSAs biosynthesis and regulation.

Species	Publication Date	Materials	Sequencing Platforms	Annotated Genes	Data Type	Reference
*P. protegens*	2022	Mycelium	Illumina Miseq	MEP pathway	Genomic Data	[2]
*D. nobile*	2024	Young leaves	Illumina Hiseq X ten	(1) Terpenoid backbone (2) TPS family (3) CYP450 family		[21]
*D. nobile*	2022	Young tenders	MGISEQ-2000	(1) TPS family (2) CYP450 family		[22]
*T. longibrachiatum*	2024	Strain UN32 treated with CoCl_2_	Illumina Hiseq4000	ROS signaling pathway	Transcriptomic Data	[28]
*T. longibrachiatum*	2022	Strain MD33 treated with MeJA	Illumina Hiseq4000	(1) Alkaloid backbone (2) P450 superfamily, methyltransferase and aminotransferase		[29]
*D. officinale*	2013	Stem	454 pyrosequencing	Alkaloid backbone		[23]
*D. nobile*	2017	Stems treated with mycorrhizal fungus	Illumina Hiseq4000	Dendrobine biosynthesis pathway		[24]
*D. officinale*	2019	Leaves treated with MeJA	Illumina Hiseq4000	(1) Alkaloid backbone (2) P450 superfamily, methyltransferase and aminotransferase		[25]
*T. longibrachiatum*	2021	MD33 with its mutant UN32	Illumina Hiseq4000	(1) Sesquiterpenoid Alkaloid backbone (2) P450 superfamily, methyltransferase and aminotransferase		[30]
*D. officinale*	2017	Leaves, stems and roots	Illumina Hiseq2500	(1) Alkaloid backbone (2) CYP450s		[26]

**Table 2:** A quality comparison of two *D. nobile* genome versions.

Items	Yunan Province [22]	Guizhou Province [21]
Sequencing platform	MGISEQ-2000 Pacbio sequel II Hi-C	Illumina Hiseq X ten Pacbio sequel II Hi-C
Genome size	1.19 Gb	1.19 Gb
Heterozygosity	1.35%	2.03%
Contig N50	1.61 Mb	10.01 Mb
Assembly level	Chromosome	Chromosome
WGD	2	0
Repetitive ratio	61.07%	42.3%
SNPs	Not reported	Not reported
Protein-coding genes	29,476	31,672
Functionally annotated genes	27,765	30,828

## Data Availability

Not applicable.
